# Characterization of M11-like and OC125-like Monoclonal Antibody Binding to CA125 Tandem Repeats

**DOI:** 10.3390/bios15100633

**Published:** 2025-09-23

**Authors:** Trisha Nair, Caitlin R. McEntee, Chien-Wei Wang, Anubhuti Srivastava, Jane C. March, Eliza K. Hanson, Rebecca J. Whelan

**Affiliations:** 1 Department of Chemistry, University of Kansas, Lawrence, KS 66045, USA; tnair02@ku.edu (T.N.); caitlinmcentee@ku.edu (C.R.M.); awayne1@gmail.com (C.-W.W.); anubhuti@ku.edu (A.S.); janemarch@ku.edu (J.C.M.); hansone@rowan.edu (E.K.H.); 2 Ralph N. Adams Institute for Bioanalytical Chemistry, University of Kansas, Lawrence, KS 66047, USA

**Keywords:** ovarian cancer, CA125, M11-like, OC125-like, antibody, immunoassay, ELISA, SPR

## Abstract

The CA125 epitope within the MUC16 tandem repeat region is detected via the CA125 II test for ovarian cancer surveillance. This test utilizes the M11 and OC125 antibodies. A revised model of MUC16 with 19 tandem repeats has recently been identified, including splice variants that exclude entire repeats. Additionally, OC125 has exhibited gaps in coverage of the tandem repeat region. To identify antibodies that bind more repeats and are suitable for spliceoform detection, more antibodies must be characterized using the revised model. This study characterized the binding of two M11-like and two OC125-like antibodies against the updated tandem repeat numbering system. 16 individual tandem repeats were expressed and purified. Binding interactions between each of the antibodies and recombinant repeats were examined by indirect enzyme-linked immunosorbent assay (ELISA) and surface plasmon resonance (SPR). The M11-like antibodies displayed different binding patterns when compared to each other, while the two OC125-like antibodies exhibited similar binding patterns. M11-like clone M77161 bound to all 16 repeats tested, indicating that it may be suitable for accurate detection of CA125. These findings demonstrate how different antibodies vary in their binding to CA125, contributing to ongoing development of improved clinical and research tools for ovarian cancer.

## 1. Introduction

Ovarian cancer continues to be the most lethal gynecologic malignancy affecting women, with 20,890 estimated new cases and 12,730 women predicted to die from the disease in 2025, making it the fifth-highest cause of cancer deaths for women residing in the United States [1]. When patients are diagnosed during Stage I, up to 90% of them can be cured, while diagnosis in Stage II is associated with a 5-year survival rate of 70% [2]. However, only 20% of ovarian cancer cases are identified during these early stages [2]. By contrast, late-stage diagnoses of ovarian cancer are associated with significantly lower survival rates at or below 20% [2]. These statistics highlight the need for improvements in early diagnostic and screening tools, which may enable the detection of ovarian cancer at earlier stages, thereby increasing survival rates.

Many clinical decisions pertaining to ovarian cancer rely on measuring serum levels of Cancer Antigen 125 (CA125) through the CA125 II test [3,4,5]. Following the development of the anti-CA125 antibody OC125 by Bast and co-workers, the first iteration of the CA125 test was constructed using OC125 in a homologous double-determinant immunoassay [6,7]. The establishment of the M11 antibody by O’Brien and co-workers then led to a second generation of CA125 tests in which M11 and OC125 were used to capture and detect CA125 [5,8]. This CA125 II test is now approved by the FDA to monitor disease progression and response to treatment for ovarian cancer [4]. Despite its frequent use in clinical settings, the mechanism by which the CA125 II test functions is still under investigation, and the test cannot be used for screening in the general population due to its limited sensitivity, specificity, and positive predictive value [3,9]. To improve the clinical utility of the CA125 II test, efforts must be undertaken to examine the mechanism by which antibodies bind to the CA125 epitope.

CA125 is a peptide epitope on the large transmembrane glycoprotein MUC16, which contains an N-terminal domain with extensive N- and O-linked glycosylation, a tandem repeat region, a C-terminal domain spanning the plasma membrane, and a cytoplasmic tail [3,10]. The precise location of the CA125 epitope remains unknown. Studies have determined that the CA125 epitope is located somewhere within the tandem repeat region of MUC16, which was originally believed to possess 63 similar, yet nonidentical sequences of 156 amino acids [11,12]. Our group has recently proposed a revised molecular model of MUC16 containing 19 tandem repeats, with each repeat consisting of ~156 amino acids; the amino acid sequence is complete conserved at some positions and highly variable at others [13]. Studies have shown that the anti-CA125 antibodies display variable binding to the tandem repeat region of MUC16, and our group recently examined how the M11 and OC125 antibodies bind to CA125 using the updated molecular model of MUC16 with 19 tandem repeats [14,15,16]. That study [16] entailed the expression of individual tandem repeat proteins R1–R17 (excluding R14), the same set of repeat proteins that is examined in the present report. The binding of clinical antibodies to these repeats was characterized by complementary methods [16]. The results of that study indicated that OC125 displays significant gaps in coverage of MUC16’s tandem repeat region, with OC125 binding to only 11 out of 16 repeats tested. This finding highlights the potential utility of alternative antibodies that bind epitopes on different proteoforms or recognize more units within the tandem repeat region, which may aid in accurate detection of CA125 and therefore improve the sensitivity of the current CA125 II blood test [16]. In addition, we have confirmed the presence of splice variants in MUC16 mRNA, which have previously been reported [13,17,18]. Some of these splice variants exclude entire tandem repeats, indicating that some proteoforms may not contain the CA125 epitopes [13].

Since the development of the M11 and OC125 antibodies, additional antibodies targeting CA125 have been developed and classified into three epitope groups based on competitive cross-inhibition studies: epitope group A (termed “OC125-like”), epitope group B (termed “M11-like”), and epitope group C (termed “OV197-like”) [19]. The M11-like and OC125-like antibodies have been observed to share binding locations with the M11 and OC125 antibodies, respectively, based on these competition studies [19]. However, many of these clones have yet to be characterized using the new MUC16 model. A more nuanced understanding of how these “-like” antibodies differ in their binding to CA125 could help identify clones that display greater coverage of the tandem repeat region than the OC125 antibody. These clones may be better suited for detection of CA125 compared to OC125. Additionally, a detailed analysis of the “-like” antibodies and their binding epitopes could aid in further investigations aiming to examine the function and use of CA125. M11-like and OC125-like antibodies have been used in a variety of studies, including investigations of CA125/mesothelin-dependent cell attachment, Siglec-8 and Siglec-9 ligand distribution and characteristics, quantification of CA125 with programmable bio-nano-chips, and phenotyping of plasma-derived exosomes using novel extracellular vesicle (EV) arrays [20,21,22,23,24,25,26]. Additionally, these antibodies have been used for histopathological analysis of patient-derived tumor organoids (PDTO) in efforts to improve chemodynamic therapy and chemotherapy [27,28,29,30]. These studies emphasize the potential utility of M11-like and OC125-like antibodies as research reagents in diverse settings. Ultimately, improved detection and examination of CA125 relies on a robust analysis of how CA125 binds to a variety of antibodies, which can allow us to selectively use different antibodies for unique research and diagnostic tools.

In this study, we characterized two M11-like (clones M002203 and M77161) and two OC125-like (clones M002201 and X306) monoclonal anti-CA125 antibodies using enzyme-linked immunosorbent assay (ELISA) and surface plasmon resonance (SPR) methods. The binding affinity between each of these antibodies and sixteen individual repeat proteins from the revised tandem repeat region of MUC16 was studied. The central hypothesis of this work is that probing each repeat individually with the “-like” antibodies using the revised molecular model will provide an accurate representation of the antibody binding patterns. This information can then enable identification of antibodies that may be suitable for detecting certain MUC16 spliceoforms or bind to more repeats in the tandem repeat region than the clinically used antibodies. These results provide additional knowledge on how different antibodies exhibit variable binding to CA125 with implications in both clinical and research use.

## 2. Materials and Methods

### 2.1. CA125 Recombinant Protein Expression and Purification

MUC16 tandem repeat sequences were obtained as described previously [13] and were cloned in pET-14b vectors (GenScript, Piscataway, NJ, USA) using BamHI and XhoI sites with a 6-His tag on the N-terminus. The plasmids were transformed in SHuffle T7 Express *E. coli* cells (New England Biolabs, Beverly, MA, USA). The *E. coli* cells were grown to the late stationary phase at 30 °C in MagicMedia^TM^ E. coli Expression Medium (Thermo, Waltham, MA, USA) containing 100 μg/mL ampicillin. Freeze-thaw cycles in liquid nitrogen, followed by sonication in lysis buffer (20 mM sodium phosphate, 10 mM imidazole, 300 mM sodium chloride) and cOmplete^TM^ protease inhibitor (Thermo, St. Louis, MO, USA) were performed to harvest and lyse the cells. The recombinant repeat proteins were then purified on HisPur^TM^ Ni-NTA Resin (Thermo, Waltham, MA, USA), followed by size-exclusion chromatography using a Superdex 75 10/300 GL column with an ÄKTA pure^TM^ 25 chromatography system. Three repeats (R14, R18, R19) were unable to be expressed in soluble form using this approach and were therefore not probed in this study. Western blotting was performed to examine the purity and molecular weight of the recombinant proteins, as described below.

### 2.2. Western Blot

Recombinant repeat proteins were separated by size via sodium dodecyl sulfate-polyacrylamide gel electrophoresis (SDS-PAGE) using a 16% acrylamide gel before being transferred to a polyvinylidene difluoride (PVDF) membrane. After transfer, the membrane was blocked with a solution of 5% non-fat milk in Tris buffered saline with 0.05% Tween-20 (TBS-T) for 1 h at room temperature (RT). The membrane was then incubated overnight at 4 °C with anti-6xHis (Thermo, 1:1000) primary antibody. After incubation, the membrane was washed with TBS-T for 1 h (4 rounds of 15 min washes). The membrane was then incubated at RT with goat anti-mouse HRP secondary antibody (Thermo, 1:10,000) for 1 h. The membrane was washed as for the primary antibody and then developed using Pierce ECL Western blotting Substrate (Thermo) and imaged with a ChemiDoc system (Bio-Rad, Hercules, CA, USA).

### 2.3. ELISA

Indirect ELISA was conducted by immobilizing the recombinant repeat proteins at 5× capacity on Pierce Nickel-Coated Plates via their 6xHis tag. 886.5 ng of each recombinant repeat was diluted in PBS before immobilization for 1 h at RT. Wells containing a protein that should not interact with CA125 antibodies (HE4) and wells containing no antigen were used as negative controls. Recombinant repeat protein–antibody combinations were tested in triplicate. After repeat immobilization, three washes with 200 µL PBS-T (0.05% Tween-20) were performed before addition of 100 µL of one of the following primary antibodies: anti-CA125 epitope group A (Biosynth, OC125-like, M002201, 1:15,600 or Biosynth, OC125-like, X306, 1:13,200) or anti-CA125 epitope group B (Biosynth, M11-like, M002203, 1:28,600 or Biosynth, M11-like, M77161, 1:22,600). The primary antibody was incubated for 1 h at RT. For experiments varying primary antibody incubation time, 100 µL PBS was initially added to each well at the beginning of the incubation period. PBS was then replaced with 100 µL primary antibody (Biosynth, M11-like, M77161, 1:22,600; Biosynth, OC125-like, M002201, 1:15,600; or Biosynth, OC125-like, X306, 1:13,200) at varied times to test the following primary antibody incubation periods: 0 min, 3 min, 25 min, and 60 min. After primary antibody incubation, three washes with 200 µL PBS-T were performed before addition of 100 µL goat anti-mouse HRP secondary antibody (Thermo, 1:20,000) followed by incubation for 1 h at RT. After a final set of three washes with 200 µL PBS-T, chemiluminescent signals were developed with SuperSignal ELISA Femto Substrate (Thermo). A SpectraMax M5 plate reader (Molecular Devices, San Jose, CA, USA) was used for detection of relative chemiluminescence units at 425 nm.

### 2.4. SPR Materials

High Refractive Index Solution (Glycerol 32%), Low Refractive Index Solution (Glycerol 8%), 10 mM HCl Conditioning Solution, 1 M Ethanolamine, 16-Channel CMD-Carboxyl Cartridges with Cartridge Fluid, and Alto Surface Plasmon Resonance Spectrometer were purchased from Nicoya Lifesciences (Kitchener, ON, Canada). PBST (0.1% *v*/*v*) was prepared in-house by adding Tween-20 to PBS from GrowCells (Irvine, CA, USA). All samples were prepared in PBS and supplemented with Tween-20 (0.1% *v*/*v*). H_2_OT was prepared in-house by adding Tween-20 to 18.2 MΩ-cm water. 1-ethyl-3-(3-dimethylaminopropyl)carbodiimide hydrochloride (EDC) in 7.7 mg aliquots and N-hydroxysuccinimide (NHS) in 4.6 mg aliquots were obtained from Nicoya Lifesciences and prepared to 200 mM in H_2_OT (0.1% *v*/*v*). Anti-His mAb aliquots (4 µL at 0.2 mg/mL stock) were prepared to 5 ug/mL in 10 mM sodium acetate (pH 4.0), both obtained from Nicoya Lifesciences. 10 mM Glycine-HCl regeneration solution (pH 1.5) was prepared in-house. CA125 mAbs were prepared to 15 ug/mL in PBST prior to loading on the cartridge. Anti-His immobilization data was analyzed using TraceDrawer Analysis 1.9.2 (Ridgeview Instruments, Uppsala, Sweden).

### 2.5. SPR Binding Kinetics Assay Sample Preparation

Immobilization of the 6-His tagged recombinant tandem repeats onto CBX CMD cartridges was conducted via capture kinetics with anti-His mAbs. All repeats were prepared to a concentration of 60 µg/mL in PBS-T (0.1%), with the exception of R17 tested with clone M002203, which was prepared to a concentration of 80 µg/mL. For repeats with low stock concentration, a dilution was made into PBS and spiked with Tween-20 to a final concentration of 0.1%. Volumes loaded onto the cartridges were suggested by Nicoya and are as follows: 65 µL for row R (reagents and analyte), 4 µL in rows A and B (reagents), 3 µL in row C (anti-His capture Ab), 2 µL in rows D–I (repeats), and 180 µL in row BF (running buffer).

### 2.6. SPR Instrumental Protocol

Experimental layouts and protocols were created using templates in the Nicosystem. Capture Kinetics assays were run with Single-Cycle Titration and Multi-Ligand settings. Cartridges were held at 25 °C. Default droplet settings were used for Startup, Normalize, Clean, and Build Surface steps. Default values were used for Kinetic Step settings with the exceptions of baseline droplet prior to analyte association (extended from 200 to 600 s), analyte dissociation (extended from 600 to 1200 s), and final baseline (1155 s).

### 2.7. SPR Binding Kinetics Assessment

The Nicosystem software (version 2.5.2, Kitchener, ON, Canada) fit the SPR data to a 1:1 Langmuir binding model, calculating and reporting values for the analyte binding signal with all occupied ligand sites (R_max_), association rate constant (k_a_), dissociation rate constant (k_d_), and apparent equilibrium dissociation constant (K_D_; avidity effects preclude us from concluding that these are the true equilibrium dissociation constant values). Binding affinity constants for each repeat-antibody pair (tested in triplicate) were averaged. For repeat–antibody pairs that displayed an outlier in the R_max_ value for one of the three rounds tested, the data pertaining to that round was excluded from analysis and the average of two rounds was taken. Interactions with R_max_ < 10 RU and binding curves that did not display an increase in signal with increases in analyte concentration were characterized as non-binding. R_max_ was calculated using the following formula: (MW analyte/MW ligand) × RL, where RL is the ligand immobilization level. For the ligand (tandem repeat protein) a MW of 19,000 Da was used. For the analyte (IgG) a MW of 150,000 Da was used.

## 3. Results

### 3.1. Western Blotting

**Figure 1:**
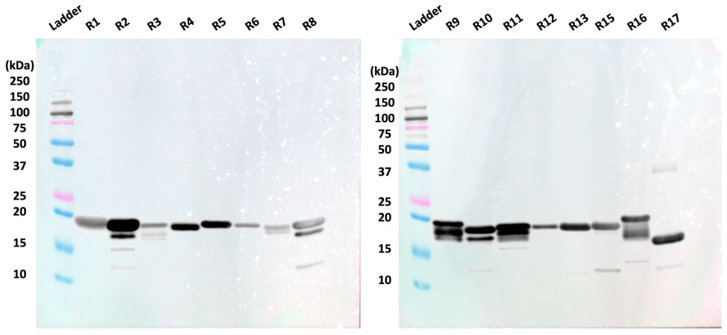
Anti-6xHis Western blot shows 15 of 16 tested recombinant repeats around 19.7 kDa. R17 is observed at a lower position on the anti-His Western blot due to its lower molecular weight (18.1 kDa).

After sixteen of the nineteen tandem repeats were expressed in *E. coli* cells and purified via Ni-NTA and size exclusion chromatography, they were separated via SDS-PAGE. A Western blot in which the repeats were probed with an anti-6xHis antibody was then conducted to confirm the presence of recombinant protein with a 6xHis tag (Figure 1). All recombinant tandem repeats were visualized on the anti-6xHis Western blot. Repeat 17 appeared lower on the membrane compared to the other repeats, consistent with its smaller molecular weight.

### 3.2. Enzyme-Linked Immunosorbent Assay (ELISA)

**Figure 2:**
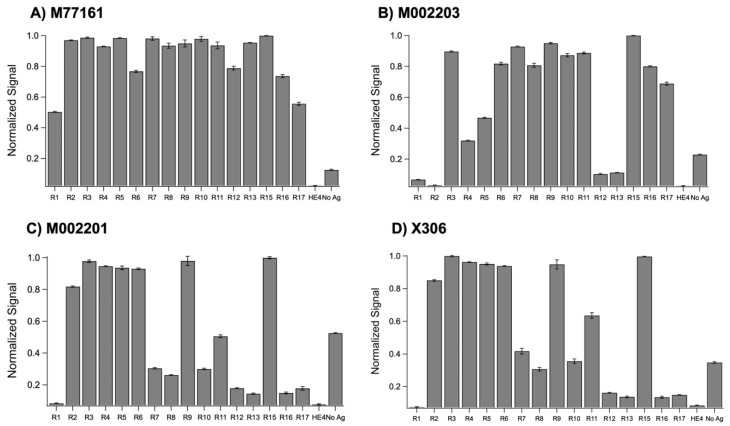
Normalized ELISA signals for recombinant repeats probed with (**A**) M77161 (M11-like), (**B**) M002203 (M11-like), (**C**) M002201 (OC125-like), and (**D**) X306 (OC125-like). Error bars are indicative of standard error of the mean (n = 3).

Indirect ELISA tests were conducted between the set of recombinant repeats and the four monoclonal antibodies in triplicate before the average, normalized signal for each repeat-antibody combination was determined and plotted (Figure 2). A threshold value of 0.2 (20% of the maximum signal) was used to categorize repeat-antibody combinations as binding or non-binding interactions, with signals above 0.2 classified as binding. (The rationale for using 20% of the maximum binding signal as a cutoff value comes from our earlier work characterizing the binding of expressed MUC16 tandem repeat proteins with OC125 and M11 [16]. In that study, when repeat–antibody interactions were tested using ELISA, low chemiluminescence signals in some cases indicated that there was weak or no binding between the repeat and the antibody. When the binding interactions were validated using SPR, the interactions that gave rise to weak ELISA signals showed no increase in SPR signal with increasing concentration of the antibody, confirming the non-binding interaction. It was observed that all such interactions had a chemiluminescent signal in ELISA < 20% of the maximum signal. A pooled *t*-test showed that the signals for the binding interactions were significantly different from non-binding. In the present study, to keep the analysis consistent with the previous study of the clinical antibodies [16], we use the same cutoff value for the ELISA results.)

A two-sample, pooled *t*-test assuming unequal variances was conducted between the repeat signals categorized as binding and the repeat signals categorized as non-binding for the following antibodies: M002203 (M11-like), M002201 (OC125-like), and X306 (OC125-like). A pooled *t*-test was not performed for clone M77161 (M11-like) since all repeat-antibody interactions were categorized as binding. Each of the three pooled *t*-tests at a 95% confidence level resulted in *p* < 0.0001, indicating a highly significant difference between the binding interactions and the non-binding interactions. The M11-like clones displayed different binding patterns, with clone M77161 binding to the most repeats out of the four antibodies tested. Clone M77161 was observed to bind to all repeats across the tandem repeat region, with weaker binding to R1 and R17. Clone M002203 showed binding to R3–R11 and R15–R17. Generally, the M11-like antibodies were found to bind to more repeats than the OC125-like antibodies. The OC125-like antibodies displayed nearly identical binding patterns, exhibiting binding to R2–R11 and R15 with weak binding for R7, R8, and R10. The no-antigen control signal was notably high for three of the antibodies tested, with signals above the threshold of 0.2.

### 3.3. Surface Plasmon Resonance (SPR)

**Figure 3:**
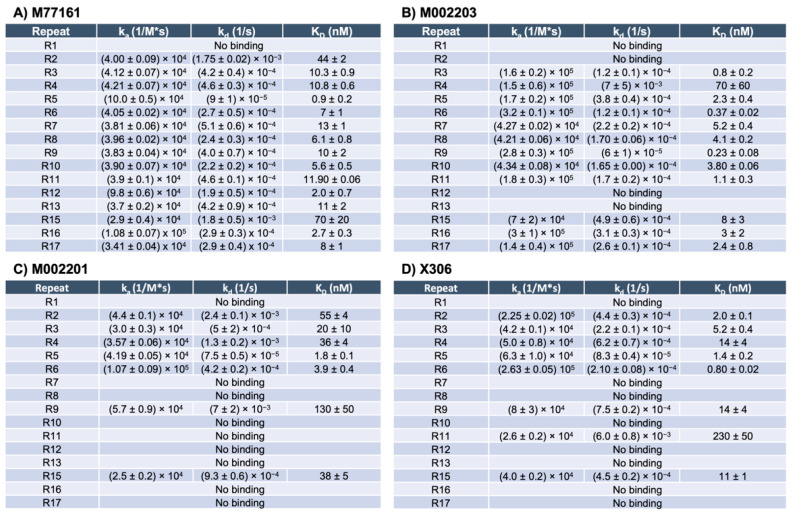
SPR binding affinity constants for M11-like clones M77161 and M002203, and OC125-like clones M002201 and X306. The constants reported are the association rate constant (k_a_), dissociation rate constant (k_d_), and the apparent equilibrium dissociation constant (K_D_). The error is given as standard error of the mean, with n = 3 for all combinations except M002203 with R4, M002201 with R9, and X306 with R11, for which n = 2.

**Figure 4:**
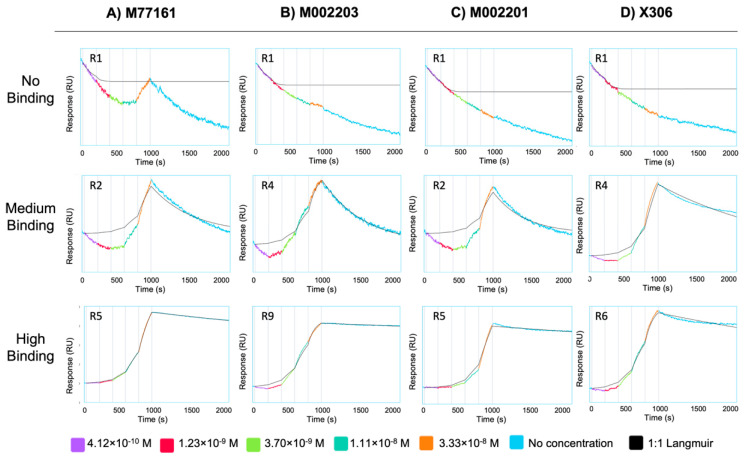
Sensorgrams depicting no binding (top row), medium binding (middle row), and high binding (bottom row) interactions for antibody clones (**A**) M77161, (**B**) M002203, (**C**) M002201, and (**D**) X306. Sensorgrams were obtained from the Nicosystem software. The repeat protein corresponding to each repeat–antibody combination is given in the top left corner of each sensorgram. The antibody concentrations used to generate the curves are provided in the legend.

SPR experiments using capture kinetics with anti-His capture antibodies were performed to obtain binding curves and binding affinity constants for each repeat-antibody interaction. Each interaction was tested in triplicate and the binding affinity constants for each interaction were averaged across three rounds, except for the following pairs: M002203 with R4, M002201 with R9, and X306 with R11. For these three interactions, the R_max_ value for one of the rounds was determined to be an outlier, and data pertaining to that round was excluded from analysis. Interactions with an R_max_ value above 10 RU and binding curves displaying an increase in signal with increases in analyte concentration were categorized as binding while all other interactions were denoted as nonbinding. For repeat-antibody combinations that were classified as binding, the association rate constant (k_a_), dissociation rate constant (k_d_), and apparent equilibrium dissociation constant (K_D_) as determined by the Nicosystem software are shown in Figure 3. Consistent with the ELISA results, the M11-like antibodies bound to more tandem repeats than the OC125-like antibodies. Clone M77161 showed binding for R2–R17 while clone M002203 had binding interactions with R3–R11 and R15–R17. Clone M002201 displayed binding to R2–R6, R9, and R15. Clone X306 exhibited binding to R2–R6, R9, R11, and R15. The binding patterns determined by SPR for the two OC125-like clones indicate a slight departure from the ELISA data since the binding patterns are not identical (clone X306 binds to R11 while clone M002201 does not). Sensorgrams showing high binding, medium binding, and nonbinding are shown for each of the four antibodies in Figure 4.

### 3.4. Comparison of Analytical Methods

**Figure 5:**
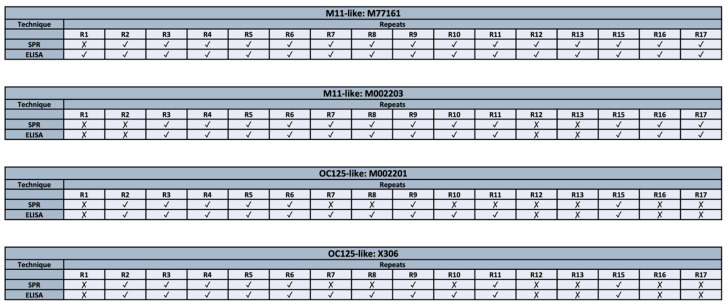
Comparison of binding patterns between two characterization techniques (ELISA and SPR) for M11-like clones (M77161 and M002203) and OC125-like clones (M002201 and X306).

A summary of the binding patterns obtained from both methods (ELISA and SPR) can be seen in Figure 5. When comparing the binding patterns obtained from the ELISA and SPR techniques, eight discrepancies are observed, corresponding to the following antibody-repeat combinations: M77161 with R1, M002201 with R7, R8, R10, and R11, and X306 with R7, R8, and R10. For all discrepancies, binding is observed in ELISA while the SPR data indicates no binding interaction. There are fifty-six combinations for which both characterization methods provide the same result. Notably, R3–R11 display binding to both M11-like antibodies using both methods. Similarly, R2 and R3 display binding to clones M77161, M002201, and X306 in both methods. Repeats that often display no binding across both characterization methods include R1, R12, and R13.

**Figure 6:**
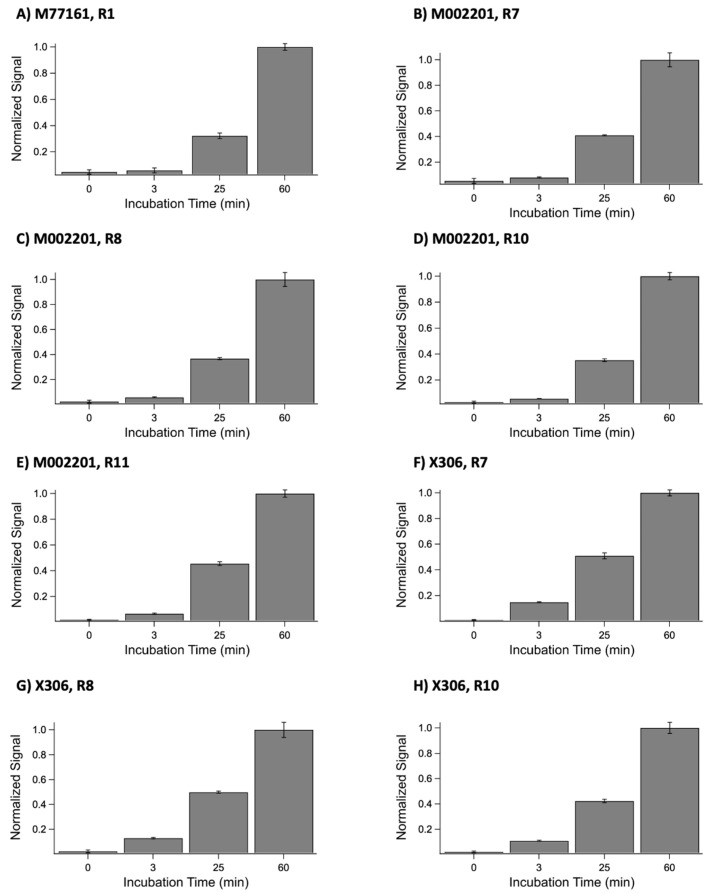
Normalized ELISA signals for the following anti-CA125 antibody and recombinant tandem repeat combinations: (**A**) M77161 with R1, (**B**) M002201 with R7, (**C**) M002201 with R8, (**D**) M002201 with R10, (**E**) M002201 with R11, (**F**) X306 with R7, (**G**) X306 with R8, and (**H**) X306 with R10. For each combination, the anti-CA125 antibody was incubated with the repeat for the following time periods: 0 min, 3 min, 25 min, and 60 min. Error bars are standard error of the mean (n = 3), with the exception of the 25 min conditions for each X306 combination. For these three conditions, the error bars are standard error of the mean (n = 2).

One potential explanation for the discrepancies between the ELISA and SPR data, in which binding occurred in the ELISA but not in SPR, could stem from the difference in contact time between the repeat and monoclonal antibody in both methods [16]. In the ELISA, the contact time between the recombinant repeat and antibody is 60 min, while the contact time between the ligand and analyte in SPR is 3 min. To test this hypothesis, indirect ELISA experiments were conducted in which the primary antibody incubation time was varied. Each repeat-antibody pair that produced a discrepancy in results between the two techniques was tested. The results from these experiments are shown in Figure 6. A one-tailed paired *t*-test at 95% confidence revealed a statistically significant difference between the 3 min condition and 60 min condition for each repeat-antibody pair tested. The *p*-values were determined to be *p* = 0.001 for M77161 with R1, *p* = 0.002 for M002201 with R7, *p* = 0.002 for M002201 with R8, *p* = 0.0004 for M002201 with R10, *p* = 0.0005 for M002201 with R11, *p* = 0.0002 for X306 with R7, *p* = 0.003 for X306 with R8, and *p* = 0.001 for X306 with R10. These results indicate that the contact time between the anti-CA125 antibody and recombinant repeat may be insufficient to display binding in SPR for certain repeat-antibody combinations. This provides an explanation for the discrepancies observed between the two characterization methods, placing more emphasis on the ELISA results for these instances.

## 4. Discussion

Western blotting was conducted with anti-His antibody to verify successful expression of the recombinant repeat proteins with the 6xHis tag. Bands for each expressed repeat are observed around 20 kDa (Figure 1), indicating the presence of these repeats. The band for repeat 17 appeared lower on the membrane than the other repeats, which has consistently been observed in prior studies and can be attributed to the shorter amino acid sequence of repeat 17 [13,16]. It is important to note that coverage of the tandem repeat region in the present study is incomplete because three of the repeats (R14, R18, and R19) could not be obtained in soluble form using our *E. coli* expression system. The missing repeats comprise ~16% of the tandem repeat region (3 of 19 repeats). The use of alternate expression systems, such as Chinese Hamster Ovary cells, SimpleCells, or insect cells, could enable the expression of these recalcitrant repeat proteins and is a strategy we are actively pursuing. Despite this modest limitation in coverage, however, we contend that the insight offered on antibody–repeat binding using the 16 of 19 repeats we can express is valuable.

Indirect ELISA was used to probe each repeat protein in its native state with the four monoclonal antibodies. The binding patterns from the ELISA tests for each antibody are shown in Figure 2. Pooled *t*-tests assuming unequal variances that were performed between the binding repeat–antibody interactions and the non-binding repeat–antibody interactions for three of the four antibodies indicated a highly significant difference between the binding repeat interactions and non-binding repeat interactions, with *p* < 0.0001 for all the *t*-tests. The ELISA binding patterns for the two M11-like clones are different, with M77161 binding to more repeats. This observation indicates that these antibodies may bind to different epitopes. When comparing the ELISA binding patterns for the two OC125-like clones, we observe nearly identical binding patterns, pointing to the possibility that these two antibodies share the same epitope. These results indicate that any of the monoclonal antibodies studied may be paired together as capture and tracer in a sandwich ELISA, with the exception of one combination: clone X306 and clone M002201. The two OC125-like ELISAs were noted to have high nonspecific binding to the Ni^2+^ plate, as indicated by the strong signal from the no-antigen control. While the reason for this is still being investigated, it is possible that a component of the antibody’s sequence allows it to bind to the Ni^2+^ plate in the absence of a blocking agent. Binding in the no-antigen control could result from available His residues in close enough proximity to engage in Ni^2+^ chelation; determination of the sequences of these antibodies would be necessary to further explore this hypothesis. Alternative capture plate design (independent of the chelate effect) and inclusion of blocking agents such as serum albumin could mitigate the observed nonspecific binding in the no-antigen controls. However, this high nonspecific binding to the plate was determined not to have a significant effect on the results because a negative control in which a blocking protein (HE4) was present consistently produced low signals.

SPR with capture kinetics was also used to probe the repeats with each of the four antibodies, providing binding affinity constants for each binding interaction. The binding patterns determined from SPR show that the M11-like antibodies bind to more repeats than the OC125-like antibodies, consistent with the ELISA results. However, unlike the ELISA binding patterns for the two OC125-like clones, there is a slight difference between the SPR binding patterns for the M002201 and X306 OC125-like clones. Clone M002201 does not show binding to R11 while clone X306 does indicate binding for this repeat. The apparent K_D_ value for X306 with R11 is 230 nM, which is the highest apparent K_D_ value that was determined across all the repeat-antibody combinations tested, indicating that this was the weakest binding interaction observed. When comparing the M11-like SPR results to the M11-like ELISA results, the SPR binding patterns again show that clone M77161 binds to more repeats than clone M002203. However, clone M002203 appears to have consistently lower apparent K_D_ values compared to clone M77161, indicating that although it may bind fewer repeats, many of its binding interactions may be stronger than those of clone M77161.

When comparing the binding patterns for each antibody between the two experimental methods, we observe some discrepancies. The discrepancy between the methods comes from observed binding in ELISA and no observed binding in SPR for the same repeat–antibody pair. Many factors may contribute to these discrepancies, including the orientation of the ligand on the surface, the ligand immobilization strategy, avidity effects, and contact time. In both ELISA and SPR experiments, the tandem repeat protein is the ligand, immobilized onto a surface through the 6xHis tag, while the antibody is added in solution as the analyte. In the case of ELISA, the chelate effect occurs when His residues interact with Ni^2+^ ions on the plate, while in the case of SPR, a covalently immobilized anti-His IgG captures the repeat via noncovalent affinity binding to the His tag. Therefore, between the two methods, the same binding partner (repeat) is immobilized, and the orientation can reasonably be expected to be the same. Surface chemistry and occupancy are uncontrolled variables in these experiments and may lead to avidity effects that differ between ELISA and SPR. However, it does not seem probable that differences in avidity could result in the observed discrepancies, in which one method (ELISA) suggested that a combination was binding while the other (SPR) suggested the combination was non-binding. We hypothesized that contact time between the repeat and antibody was the most likely contributor to observed methodological differences, since ELISA uses a contact time of 60 min while SPR uses a contact time of 3 min (this short contact time in SPR is largely set by limitations in the data buffering capacity of the instrument control software). To test this hypothesis, indirect ELISA experiments varying anti-CA125 antibody incubation time were conducted with each antibody–repeat pair that displayed a discrepancy between the characterization methods. A one-tailed paired *t*-test at 95% confidence between the 3 min and 60 min signals for each pair produced a *p* value below 0.05, indicating a statistically significant difference between the conditions. These results are consistent with our prior study examining the interactions of expressed MUC16 tandem repeat proteins with OC125 and M11 [16]. Based on these results, we believe that the shorter contact time in SPR may provide insufficient time to observe a binding interaction for certain repeat–antibody combinations. In these cases, the binding characterization obtained from ELISA takes precedence over the classification given by SPR.

In our previous study examining the binding patterns of the clinically used M11 and OC125 antibodies, we found that M11 exhibited binding to all 16 repeats tested, while OC125 only displayed binding to 11 out of 16 repeats [16]. This indicates that OC125 may not be suitable for accurate detection of CA125, as it exhibits significant gaps in coverage when used to probe CA125. Alternatively, the M11-like clone M77161 examined in this study demonstrated similar coverage to M11, binding to all repeats within the tandem repeat region that were tested. Consequently, the M77161 clone may be better suited for CA125 detection in a clinical setting as it is able to recognize more epitopes than OC125, potentially reducing false negative results and improving sensitivity. The primary objective of both the previous study [16] and the present study was the determination of a “yes/no” classification of binding. Moreover, the design of SPR experiments performed to characterize OC125 and M11 binding with expressed repeats did not enable accurate determination of apparent K_D_ values in all cases, since a software default value was used to estimate extremely slow dissociation rate constants for which an experimental value could not be determined. However, a comparison of the binding of M11 and M11-like clone M77161 can be made. Among the repeat–M11 combinations for which apparent K_D_ values were confidently determined, apparent K_D_ values ranged from a minimum of 0.13 nM to a maximum of 16.5 nM [16]. Among repeat–M77161 combinations, apparent K_D_ values range from a minimum of 0.9 nM to a maximum of 70 nM. The apparent equilibrium dissociation constants for the M11-like clone M77161 and M11 are all within the same order of magnitude, as well as displaying essentially identical patterns of binding.

When comparing the binding patterns of the OC125-like clones and OC125, we observe that the OC125-like clones bind to a similar number of repeats as the OC125 antibody, with OC125 binding to one extra repeat [16]. However, there are differences in which repeats they bind to, with repeats in the middle of the tandem repeat region more commonly having binding interactions with both OC125-like and OC125 antibodies. These results may provide insights for accurate detection of MUC16 splice variants. Many studies have reported the presence of MUC16 splice variants [17,18]. Our own studies have shown evidence of splice variants in which entire subdomains of the MUC16 tandem repeat region are not present [13]. Taken with the observation that different anti-CA125 antibodies exhibit variable binding to repeats within this tandem repeat region, it is possible that some antibodies may bind to epitopes not expressed on certain MUC16 spliceoforms, further contributing to false negatives. Therefore, an examination of antibody binding to CA125 on the level of individual repeats can help identify antibodies that are suitable for detection of different spliceoforms.

## 5. Conclusions

While prior work has characterized the binding patterns of the antibodies used in the CA125 II test (M11 and OC125), the binding interactions of many M11-like and OC125-like clones have not been well studied. Here we report the binding interactions of two M11-like clones (M002203 and M77161) and two OC125-like clones (M002201 and X306) with 16 of the 19 tandem repeats in MUC16. The repeats used in this study were based on our group’s recently published revised model of MUC16 containing 19 tandem repeats; this model supplants a previously accepted model that described MUC16 as containing 63 tandem repeats. Using indirect ELISA and SPR affinity characterization methods, binding patterns for the M11-like clones differed in number of repeats recognized, indicating that the M11-like antibodies may bind to distinct tandem repeats on MUC16 despite exhibiting similar binding characteristics. Conversely, both methods resulted in binding patterns for the OC125-like clones that were similar, indicating that these antibodies may share the same epitope. Furthermore, the M77161 clone exhibited binding to all 16 repeats tested in this study. Previous work has demonstrated that the OC125 antibody only binds to 11 of these 16 repeats, suggesting that clone M77161 may be better suited for detection of CA125 in clinical assays [16]. These findings provide a closer look at how the M11-like and OC125-like antibodies differ in their recognition of MUC16, which may assist in identification of antibodies that are more appropriate for various examinations of CA125 in both clinical and research settings. Next steps for further antibody characterization include reversing the identity of ligand and analyte, so that the antibody is the ligand and the repeat protein is the analyte. This approach would give true rate constants and K_D_ values. Further validation steps required before clinical implementation include characterization of different combinations of antibodies as capture and tracer; testing intact MUC16 from diverse sources (cell lines, pooled ascites, and individual patient samples); and the determination of figures of merit including limit of detection, linearity on dilution, intra- and inter-day variability, variability across users, and shelf stability.

## Data Availability

The original data presented in the study are openly available in KU ScholarWorks at https://hdl.handle.net/1808/36153 (accessed on 22 September 2025); or https://doi.org/10.17161/1808.36153.

## References

[1] Siegel R.L., Kratzer T.B., Giaquinto A.N., Sung H., Jemal A. (2025). Cancer statistics, 2025. CA Cancer J. Clin..

[2] Elias K.M., Guo J., Bast R.C. (2018). Early Detection of Ovarian Cancer. Hematol. Oncol. Clin. N. Am..

[3] Charkhchi P., Cybulski C., Gronwald J., Wong F.O., Narod S.A., Akbari M.R. (2020). CA125 and Ovarian Cancer: A Comprehensive Review. Cancers.

[4] Ghose A., McCann L., Makker S., Mukherjee U., Gullapalli S.V.N., Erekkath J., Shih S., Mahajan I., Sanchez E., Uccello M. (2024). Diagnostic biomarkers in ovarian cancer: Advances beyond CA125 and HE4. Ther. Adv. Med. Oncol..

[5] Kenemans P., van Kamp G.J., Oehr P., Verstraeten R.A. (1993). Heterologous double-determinant immunoradiometric assay CA 125 II: Reliable second-generation immunoassay for determining CA 125 in serum. Clin. Chem..

[6] Bast R.C., Feeney M., Lazarus H., Nadler L.M., Colvin R.B., Knapp R.C. (1981). Reactivity of a monoclonal antibody with human ovarian carcinoma. J. Clin. Investig..

[7] Bast R.C., Klug T.L., St. John E., Jenison E., Niloff J.M., Lazarus H., Berkowitz R.S., Leavitt T., Griffiths C.T., Parker L. (1983). A radioimmunoassay using a monoclonal antibody to monitor the course of epithelial ovarian cancer. N. Engl. J. Med..

[8] O’BRien T.J., Raymond L.M., Bannon G.A., Ford D.H., Hardardottir H., Miller F.C., Quirk J.G. (1991). New monoclonal antibodies identify the glycoprotein carrying the CA 125 epitope. Am. J. Obstet. Gynecol..

[9] Lee J., Nair N. (2021). The Current and Future States of Screening in Gynecologic Cancers. Obstet. Gynecol. Clin. N. Am..

[10] Felder M., Kapur A., Gonzalez-Bosquet J., Horibata S., Heintz J., Albrecht R., Fass L., Kaur J., Hu K., Shojaei H. (2014). MUC16 (CA125): Tumor biomarker to cancer therapy, a work in progress. Mol. Cancer.

[11] O’Brien T.J., Beard J.B., Underwood L.J., Dennis R.A., Santin A.D., York L. (2001). The CA 125 gene: An extracellular superstructure dominated by repeat sequences. Tumour Biol..

[12] Nustad K., Bast R.C., Brien T.J., Nilsson O., Seguin P., Suresh M.R., Saga T., Nozawa S., Børmer O.P., de Bruijn H.W. (1996). Specificity and affinity of 26 monoclonal antibodies against the CA 125 antigen: First report from the ISOBM TD-1 workshop. International Society for Oncodevelopmental Biology and Medicine. Tumour Biol..

[13] Wang C.W., Weaver S.D., Boonpattrawong N., Schuster-Little N., Patankar M., Whelan R.J. (2024). A Revised Molecular Model of Ovarian Cancer Biomarker CA125 (MUC16) Enabled by Long-read Sequencing. Cancer Res. Commun..

[14] Bressan A., Bozzo F., Maggi C.A., Binaschi M. (2013). OC125, M11 and OV197 epitopes are not uniformly distributed in the tandem-repeat region of CA125 and require the entire SEA domain. Dis. Markers.

[15] Wang C.W., Hanson E.K., Minkoff L., Whelan R.J. (2023). Individual recombinant repeats of MUC16 display variable binding to CA125 antibodies. Cancer Biomark..

[16] Wang C.-W., Srivastava A., Hanson E.K., McEntee C.R., Nair T., March J.C., Whelan R.J. (2025). Mapping the Binding Sites of CA125-Specific Antibodies on a Revised Molecular Model of MUC16. Cancers.

[17] Bouanene H., Miled A. (2010). Conflicting views on the molecular structure of the cancer antigen CA125/MUC16. Dis. Markers.

[18] Haridas D., Ponnusamy M.P., Chugh S., Lakshmanan I., Seshacharyulu P., Batra S.K. (2014). MUC16: Molecular analysis and its functional implications in benign and malignant conditions. FASEB J..

[19] Nap M., Vitali A., Nustad K., Bast R.C., O’Brien T.J., Nilsson O., Seguin P., Suresh M.R., Børmer O.P., Saga T. (1996). Immunohistochemical characterization of 22 monoclonal antibodies against the CA125 antigen: 2nd report from the ISOBM TD-1 Workshop. Tumour Biol..

[20] Scholler N., Garvik B., Hayden-Ledbetter M., Kline T., Urban N. (2007). Development of a CA125-mesothelin cell adhesion assay as a screening tool for biologics discovery. Cancer Lett..

[21] Yu H., Gonzalez-Gil A., Wei Y., Fernandes S.M., Porell R.N., Vajn K., Paulson J.C., Nycholat C.M., Schnaar R.L. (2017). Siglec-8 and Siglec-9 binding specificities and endogenous airway ligand distributions and properties. Glycobiology.

[22] Raamanathan A., Simmons G.W., Christodoulides N., Floriano P.N., Furmaga W.B., Redding S.W., Lu K.H., Bast R.C., McDevitt J.T. (2012). Programmable bio-nano-chip systems for serum CA125 quantification: Toward ovarian cancer diagnostics at the point-of-care. Cancer Prev. Res..

[23] Jorgensen M., Baek R., Pedersen S., Sondergaard E.K., Kristensen S.R., Varming K. (2013). Extracellular Vesicle (EV) Array: Microarray capturing of exosomes and other extracellular vesicles for multiplexed phenotyping. J. Extracell. Vesicles.

[24] Jorgensen M.M., Baek R., Varming K. (2015). Potentials and capabilities of the Extracellular Vesicle (EV) Array. J. Extracell. Vesicles.

[25] Jakobsen K.R., Paulsen B.S., Baek R., Varming K., Sorensen B.S., Jorgensen M.M. (2015). Exosomal proteins as potential diagnostic markers in advanced non-small cell lung carcinoma. J. Extracell. Vesicles.

[26] Baek R., Varming K., Jorgensen M.M. (2016). Does smoking, age or gender affect the protein phenotype of extracellular vesicles in plasma?. Transfus. Apher. Sci..

[27] Adeel M., Parisi S., Mauceri M., Asif K., Bartoletti M., Puglisi F., Caligiuri I., Rahman M., Canzonieri V., Rizzolio F. (2021). Self-Therapeutic Cobalt Hydroxide Nanosheets (Co(OH)(2) NS) for Ovarian Cancer Therapy. ACS Omega.

[28] Asif K., Adeel M., Rahman M., Caligiuri I., Perin T., Cemazar M., Canzonieri V., Rizzolio F. (2023). Iron nitroprusside as a chemodynamic agent and inducer of ferroptosis for ovarian cancer therapy. J. Mater. Chem. B.

[29] Asif K., Adeel M., Rahman M., Sfriso A.A., Bartoletti M., Canzonieri V., Rizzolio F., Caligiuri I. (2024). Silver nitroprusside as an efficient chemodynamic therapeutic agent and a peroxynitrite nanogenerator for targeted cancer therapies. J. Adv. Res..

[30] Asif K., Rahman M.M., Canzonieri V., Caligiuri I., Rizzolio F., Adeel M. (2025). Self-targeted nanosystem for enhanced chemodynamic cancer therapy. Biomater. Sci..

